# Free-text analysis of general practice out-of-hours (GPOOH) use by people with advanced cancer: an analysis of coded and uncoded free-text data

**DOI:** 10.3399/BJGP.2022.0084

**Published:** 2023-01-24

**Authors:** Sarah EE Mills, Alana Brown-Kerr, Deans Buchanan, Peter T Donnan, Blair H Smith

**Affiliations:** School of Medicine, University of St Andrews, St Andrews.; NHS Tayside, Ninewells Hospital, Dundee.; NHS Tayside, Ninewells Hospital, Dundee.; Population Health and Genomics Division, University of Dundee Medical School, Ninewells Hospital and Medical School, Dundee.; Population Health and Genomics Division, University of Dundee Medical School, Ninewells Hospital and Medical School, Dundee.

**Keywords:** after-hours care, cancer, general practice, pain, palliative care, terminal care

## Abstract

**Background:**

People with advanced cancer frequently use the GP out-of-hours (GPOOH) service. Considerable amounts of routine GPOOH data are uncoded. Therefore, these data are omitted from existing healthcare datasets.

**Aim:**

To conduct a free-text analysis of a GPOOH dataset, to identify reasons for attendance and care delivered through GPOOH to people with advanced cancer.

**Design and setting:**

An analysis of a GPOOH healthcare dataset was undertaken. It contained all coded and free- text information for 5749 attendances from a cohort of 2443 people who died from cancer in Tayside, Scotland, from 2013–2015.

**Method:**

Random sampling methods selected 575 consultations for free-text analysis. Each consultation was analysed by two independent reviewers to determine the following: assigned presenting complaints; key and additional palliative care symptoms recorded in free text; evidence of anticipatory care planning; and free-text recording of dispensed medications. Inter-rater reliability concordance was established through Kappa testing.

**Results:**

More than half of all coded reasons for attendance (*n* = 293; 51.0%) were ‘other’ or ‘missing’. Free-text analysis demonstrated that nearly half (*n* = 284; 49.4%) of GPOOH attendances by people with advanced cancer were for pain or palliative care. More than half of GPOOH attendances (*n* = 325; 56.5%) recorded at least one key or additional palliative care symptom in free text, with the commonest being breathlessness, vomiting, cough, and nausea. Anticipatory care planning was poorly recorded in both coded and uncoded records. Uncoded medications were dispensed in more than one- quarter of GPOOH consultations.

**Conclusion:**

GPOOH delivers a substantial amount of pain management and palliative care, much of which is uncoded. Therefore, it is unrecognised and under-reported in existing large healthcare data analyses.

## INTRODUCTION

A profound and sustained change in the delivery of palliative and end-of-life care in the UK has occurred as a result of the COVID-19 pandemic.^1^^–^^4^ Not only did the pandemic cause a substantial number of excess deaths, but also the majority of these deaths occurred outside of hospitals in care homes and other community settings.^1^^–^^3^ People dying from cancer were among those most likely to have their end- of-life care delivered in the community; in non- pandemic conditions, they would have received hospital care.^1^^,^^2^

As well as shifting the location of cancer deaths into the community, the COVID- 19 pandemic has created a large rise in late cancer diagnoses, which threatens to dramatically increase the number of people dying from cancer over the next few years. Even before COVID-19, cancer accounted for one in three deaths in the UK, and the number of people dying from cancer was increasing year on year, as cancer incidence outpaced improvements in survivability.^5^^–^^7^ COVID-19 has had a catastrophic impact on cancer survivability.^8^ The increased pressure on the health service, pausing of screening services, hesitancy of patients to seek medical attention, reduction in imaging and diagnostic services, and interruption of cancer care pathways have created a perfect storm of missed diagnoses and delayed treatments, which will result in a dramatic increase in the number of people dying from cancer over the next few years.^8^^–^^12^ People’s care will largely be delivered in the community, and, when the need for care occurs outside core hours, it will be delivered through the general practice out-of-hours (GPOOH) service. Understanding how primary palliative care is delivered to people dying from cancer has never been more urgent than now.

People dying from cancer frequently attend the GPOOH service in their last year of life.^13^^–^^16^ However, all large-scale research into unscheduled care in the UK has relied on routine healthcare datasets,^13^^,^^15^ which only contain coded data. Data coding is the process by which medical, social, prescribing, and other routinely collected clinical information are recorded in a set of predetermined categories. The information that can be gleaned from coded healthcare data is only as complete as the coding itself. The poor quality of coding of GPOOH data is a significant limiting factor in primary unscheduled care research.^13^ Previous research using coded data demonstrated that one in ten GPOOH consultations for people with advanced cancer are for pain (10.5%) and that nearly one-quarter (24.2%) are for palliative care; however, this research has also demonstrated that up to half of these consultations have missing codes, or are coded as ‘other’.^14^ Furthermore, while codes such as ‘pain’ and ‘palliative care’ can be useful in assessing the general reason for GPOOH attendance, they do not exhaustively describe the full range of symptoms being addressed in the consultation. Much of the clinical care provided by GPOOH to people with advanced cancer is therefore not assessable using coded data alone.

**Table:** How this fits in

Previous research conducted on coded healthcare data suggested that, for people with advanced cancer, one-tenth of attendances at GPOOH were for pain and nearly one-quarter were for palliative care. However, because nearly half of GPOOH consultations contain uncoded clinical data, previous studies have substantially underestimated the magnitude and complexity of palliative care and pain management being delivered in GPOOH to people dying from cancer. Free-text analysis indicates that, in GPOOH, pain is the primary reason for attendance in 28.2% (*n* = 162) of consultations, palliative care is the primary reason for attendance in 21.2% (*n* = 122) of consultations, and that palliative care symptoms are present in 56.5% (*n* = 325) of consultations. Clinicians and managers should recognise the enormity and complexity of palliative care being delivered through the GPOOH service. Managers also need to ensure there are adequate resources, support, and training available to clinicians to meet the complex needs of patients with advanced cancer in a GPOOH setting.

To the authors’ knowledge, this study reports the first analysis of UK GPOOH consultations using both coded and uncoded GPOOH data. It provides a more complete understanding of what causes patients with advanced cancer to present to GPOOH, and what medical care, including anticipatory and palliative care, is delivered to them during GPOOH attendances.

## METHOD

This retrospective cohort study gathered coded and free-text data during a 30-month period from 2013–2015 at NHS Tayside. Data were from 5749 GPOOH attendances made by a cohort of 2443 people in their last year of life before they died from cancer. Each GPOOH attendance was given a clinical code, which was assigned by the clinician at the patient’s time of attendance. For this analysis, 575 attendances (10% of the cohort’s total attendances) were selected, using the SPSS (version 22) random sample generation function. The free-text information from the clinical notes for these consultations was anonymised, stored, and analysed securely in the Safe Haven platform of the Health Informatics Centre (HIC) at the University of Dundee.

The free text that was recorded with each consultation was read and analysed by two independent reviewers (first and second author). The first author is an academic GP who also works as a GP partner and in GPOOH, and the second author is a palliative care physician. From the free text for each consultation, the reviewers determined an ‘assigned presenting complaint’, which was defined as the clinical code that most closely reflected the patient’s main reason for attendance. Reviewers also identified whether there were any palliative care symptoms recorded in the free text. Palliative care symptoms were divided into ‘key palliative care symptoms’ and ‘additional palliative care symptoms’. ‘Key palliative care symptoms’ were determined using literature review^17^^–^^27^ and included the following: anxiety; breathlessness; confusion; constipation; cough; diarrhoea; fatigue; fever; low mood; nausea; respiratory secretions; vomiting; and weakness. The term ‘additional palliative care symptoms’ was used to identify any other symptoms that the reviewers considered to be related to palliative care, and which were described in the consultation free text. Owing to its prevalence and importance, pain was considered separately from other palliative care symptoms. Thus, if a patient presented with multiple palliative care needs, for example, vomiting and breathlessness, their consultation could have ‘palliative care’ as an assigned presenting complaint, with both ‘vomiting’ and ‘breathlessness’ included as key palliative care symptoms. However, if a patient presented with breathlessness only then their consultation would be given an assigned presenting complaint of ‘breathlessness’. To avoid double-counting presentations, symptoms were only coded once (for example, in a consultation with an assigned presenting complaint of ‘breathlessness’, ‘breathlessness’ would not also be coded as a key palliative care symptom).

Reviewers also coded the presence or absence of palliative care documentation and provisions, including electronic Key Information Summary (eKIS), Do Not Attempt Cardiopulmonary Resuscitation (DNACPR) forms, and Just in Case (JIC) medications (Box 1), as well as whether any medications were recorded in the free text as being directly dispensed by GPOOH. Kappa inter-rater reliability tests were used to determine reviewer concordance for all assigned values. Full details can be found in the supplementary material for medication categorisation (see Supplementary Table S1) and Kappa analysis (see Supplementary Table S2).^28^

**Box 1.** Anticipatory care planning measures and documentation used in Scotland

**Electronic Key Information Summary (eKIS):** an electronic record that contains key palliative and end-of- life care decision-making information and statements concerning the patient’s wishes. **Do Not Attempt Cardiopulmonary Resuscitation (DNACPR) forms:** a form that advises medical and emergency care practitioners that cardiopulmonary resuscitation should not be given to a patient in the event of cardiac arrest. **Just in Case (JIC) medications:** anticipatory palliative care medication, provided in ‘JIC boxes’ that contain strong opioids and other medication for acute symptomatic relief of common palliative care symptoms, including breathlessness, agitation, and respiratory secretions. JIC medications can be prescribed by any doctor. JIC boxes typically contain medication that can be delivered by subcutaneous or intramuscular injection, and include a strong opioid, an anti-emetic, an anxiolytic, and an antimuscarinic. JIC medications are different from ‘breakthrough analgesia’, which is typically in the form of fast-acting strong opioids used for relief of pain.

## RESULTS

All assigned variables used in this analysis showed moderate (*n* = 6) or substantial (*n* = 11) inter-rater agreement.

### Assigned presenting complaints

More than half of the analysed GPOOH consultations (*n* = 293; 51.0%) had presenting complaints originally coded as ‘missing’ or ‘other’. On free-text analysis, just under half (*n* = 284; 49.4%) of all attendances had assigned presenting complaints of palliative care (*n* = 122; 21.2%) or pain (*n* = 162; 28.2%).

In consultations with presenting complaints originally coded as ‘other’ and ‘missing’, the commonest assigned presenting complaints were ‘palliative care’ and ‘pain’ (Table 1). In consultations originally coded as ‘other’ the next most frequently assigned presenting complaints were for abnormal blood results (*n* = 14; 8.6%), medication requests (*n* = 10; 6.1%), breathlessness (*n* = 8; 4.9%), and infection (*n* = 7; 4.3%). In consultations with missing presenting complaint codes, the next most frequent assigned presenting complaints were nursing care (for example, catheter problems) (*n* = 24; 18.5%), breathlessness (*n* = 8; 6.2%), confusion (*n* = 6; 4.6%), and failed encounters (*n* = 6; 4.6%). Among the 575 attendances analysed, one in four (*n* = 141; 24.5%) were originally coded as ‘palliative care’ (*n* = 101; 17.6%) or ‘pain’ (*n* = 40%; 7.0%).

**Table 1.** Original and assigned presenting-complaint clinical codes for attendances included in the free-text analysis^a^

**Clinical codes: main reason for attendance**	**Original presenting complaint, *n* (%)**	**Assigned presenting complaints, *n* (%)**				

	**All analysed attendances (*n*= 575)**	**All analysed attendances (*n* = 575)**	**Attendances originally coded as ‘Other’ (*n*= 163)**	**Attendances originally coded as ‘Missing’ (*n* = 130)**	**Attendances originally coded as ‘Pain’ (*n* = 40)**	**Attendances originally coded as ‘Palliative care’ (*n* = 101)**
**Palliative care**	101 (17.6)	122 (21.2)	34 (20.9)	10 (7.7)	0 (0.0)	72 (71.3)

**Pain and pain-related:**	40 (7.0)	162 (28.2)	47 (28.8)	32 (24.6)	38 (95.0)	19 (18.8)
Pain (general)	7 (1.2)	76 (13.2)	22 (13.5)	17 (13.1)	16 (40.0)	14 (13.9)
Abdominal pain	18 (3.1)	51 (8.9)	12 (7.4)	6 (4.6)	12 (30.0)	5 (5.0)
Chest pain	4 (0.7)	21 (3.7)	7 (4.3)	6 (4.6)	6 (15.0)	0 (0.0)
Back pain	6 (1.0)	6 (1.0)	5 (3.1)	0 (0.0)	1 (2.5)	0 (0.0)
Headache	3 (0.5)	6 (1.0)	0 (0.0)	2 (1.5)	3 (7.5)	0 (0.0)
Musculoskeletal	2 (0.3)	2 (0.3)	1 (0.6)	1 (0.8)	0 (0.0)	0 (0.0)

**Lower respiratory tract infection**	31 (5.4)	32 (5.6)	4 (2.5)	1 (0.8)	0 (0.0)	1 (1.0)

**Breathlessness**	13 (2.3)	30 (5.2)	8 (4.9)	8 (6.2)	0 (0.0)	2 (2.0)

**Medication request**	27 (4.7)	30 (5.2)	10 (6.1)	2 (1.5)	0 (0.0)	0 (0.0)

**Nursing care (for example, catheter problems)**	2 (0.3)	25 (4.3)	1 (0.6)	24 (18.5)	0 (0.0)	0 (0.0)

**Vomiting**	18 (3.1)	23 (4.0)	3 (1.8)	1 (0.8)	0 (0.0)	2 (2.0)

**Abnormal blood results^b^**	0 (0.0)	19 (3.3)	14 (8.6)	0 (0.0)	0 (0.0)	2 (2.0)

**Other infection (including sepsis)**	4 (0.7)	16 (2.8)	7 (4.3)	2 (1.5)	0 (0.0)	0 (0.0)

**Fall**	0 (0.0)	10 (1.7)	6 (3.7)	2 (1.5)	1 (2.5)	0 (0.0)

**Syncope or collapse**	0 (0.0)	9 (1.6)	5 (3.1)	4 (3.1)	0 (0.0)	1 (1.0)

**Urinary tract infection**	5 (0.9)	9 (1.6)	1 (0.6)	3 (2.3)	1 (2.5)	0 (0.0)

**Confusion**	2 (0.3)	8 (1.4)	0 (0.0)	6 (4.6)	0 (0.0)	0 (0.0)

**Dizziness**	5 (0.9)	7 (1.2)	3 (1.8)	1 (0.8)	0 (0.0)	0 (0.0)

**Failed encounter**	1 (0.2)	7 (1.2)	1 (0.6)	6 (4.6)	0 (0.0)	0 (0.0)

**Stroke or TIA**	2 (0.3)	7 (1.2)	1 (0.6)	3 (2.3)	0 (0.0)	2 (2.0)

**Death**	0 (0.0)	6 (1.0)	1 (0.6)	5 (3.8)	0 (0.0)	0 (0.0)

**Upper respiratory tract infection**	1 (0.2)	5 (0.9)	1 (0.6)	1 (0.8)	0 (0.0)	0 (0.0)

**‘Other’**	163 (28.3)	7 (1.2)	5 (3.1)	2 (1.5)	0 (0.0)	0 (0.0)

**Missing**	130 (22.6)	—	—	—	—	—

**Aggregated data for other clinical codes^a^**	30 (5.2)	41 (7.1)	11 (6.7)	17 (13.1)	0 (0.0)	0 (0.0)

a *Aggregated data for assigned presenting-complaint clinical codes, which were allocated to<5 attendances included in free-text analysis: haematuria, agitation, constipation, cough, epistaxis, haematemesis, weakness, drowsiness, diarrhoea, medication overdose, medication error, urinary retention, acute neurological symptoms, anxiety, choking, jaundice, leaking stent, nursing care, PR bleed, reflux, seizure, stoma problems, and wound care.*

b *‘Abnormal blood results’ describes the results of haematological investigations undertaken in core hours, but where the results were identified out of hours and required urgent or immediate action from GP out of hours. PR = per rectum. TIA = transient ischaemic attack.*

### Key palliative care symptoms and additional palliative care symptoms

In total, 325 (56.5%) of the 575 consultations analysed at free text contained ≥1 key or additional palliative care symptom. Key palliative care symptoms were recorded in the free text in half (*n* = 288 attendances; 50.1%) of all consultations analysed. In consultations originally coded as ‘other’ or ‘missing’, 46.0% (*n* = 75) and 30.8% (*n* = 40) of attendances, respectively, recorded at least one key palliative care symptom in their free text (data not shown). Breathlessness was the commonest key palliative care symptom recorded in free text, followed by vomiting, cough, and nausea (Table 2). Fatigue, anorexia, and low mood were relatively over-represented in consultations originally coded as ‘other’. For all key palliative care symptoms, attendances originally coded as ‘missing’ had a lower percentage of attendances that recorded key palliative care symptoms than the cohort attendance average. In consultations originally coded as pain, the most recorded key palliative care symptoms were nausea (*n* = 8; 20.0%), vomiting (*n* = 7; 17.5%), breathlessness (*n* = 6; 15.0%), constipation (*n* = 5; 12.5%), and cough (*n* = 5; 12.5%). In consultations originally coded as palliative care, the most frequently recorded key palliative care symptoms were breathlessness (*n* = 21; 20.8%), vomiting (*n* = 21; 20.8%), nausea (*n* = 15; 14.9%), fatigue (*n* = 14; 13.9%), and respiratory secretions (*n* = 12; 11.9%).

**Table 2.** Key and additional palliative care symptoms recorded in free text

**Symptoms**	**Incidence in all analysed free text (*n* = 575 attendances), *n* (%)**	**Incidence in attendances originally coded as ‘Other’ (*n* = 163), *n* (%)**	**Incidence in attendances originally coded as ‘Missing’ (*n* = 130), *n* (%)**	**Incidence in attendances originally coded as ‘Pain’ (*n* = 40), *n* (%)**	**Incidence in attendances originally coded as ‘Palliative care’ (*n* = 101), *n* (%)**
**Key palliative care symptoms recorded in free text**					
Breathlessness	116 (20.2)	29 (17.8)	18 (13.8)	6 (15.0)	21 (20.8)
Vomiting	84 (14.6)	22 (13.5)	13 (10.0)	7 (17.5)	21 (20.8)
Cough	73 (12.7)	18 (11.0)	7 (5.4)	5 (12.5)	10 (9.9)
Nausea	65 (11.3)	8 (4.9)	4 (3.1)	8 (20.0)	15 (14.9)
Weakness	52 (9.0)	5 (3.1)	0 (0.0)	2 (5.0)	8 (7.9)
Fatigue	46 (8.0)	17 (10.4)	4 (3.1)	2 (5.0)	14 (13.9)
Anorexia	45 (7.8)	16 (9.8)	3 (2.3)	4 (10.0)	8 (7.9)
Confusion	44 (7.7)	13 (8.0)	9 (6.9)	2 (5.0)	10 (9.9)
Fever	36 (6.3)	7 (4.3)	4 (3.1)	2 (5.0)	5 (5.0)
Respiratory secretions	36 (6.3)	17 (10.4)	7 (5.4)	2 (5.0)	12 (11.9)
Constipation	31 (5.4)	5 (3.1)	5 (3.8)	5 (12.5)	5 (5.0)
Anxiety	26 (4.5)	11 (6.7)	0 (0.0)	0 (0.0)	8 (7.9)
Diarrhoea	18 (3.1)	2 (1.2)	2 (1.5)	1 (2.5)	5 (5.0)
Low mood	7 (1.2)	15 (9.2)	3 (2.3)	0 (0.0)	1 (1.0)
**Additional palliative care symptoms recorded in free text**					
Agitation or distress	24 (4.2)	3 (1.8)	3 (2.3)	0 (0.0)	13 (12.9)
Oedema	10 (1.7)	1 (0.6)	0 (0.0)	1 (2.5)	7 (6.9)
Weight loss or cachexia	9 (1.6)	4 (2.5)	0 (0.0)	1 (2.5)	2 (2.0)
Dizziness	8 (1.4)	1 (0.6)	2 (1.5)	1 (2.5)	2 (2.0)
Jaundice	6 (1.0)	1 (0.6)	0 (0.0)	0 (0.0)	2 (2.0)
Coma or unconscious	5 (0.9)	0 (0.0)	2 (1.5)	0 (0.0)	1 (1.0)
Reflux or dysphagia	5 (0.9)	2 (1.2)	0 (0.0)	1 (2.5)	0 (0.0)
Acute neurology	4 (0.7)	0 (0.0)	4 (3.1)	0 (0.0)	0 (0.0)
Hallucinations	4 (0.7)	1 (0.6)	1 (0.8)	0 (0.0)	1 (1.0)
PR or PV bleeding	4 (0.7)	0 (0.0)	1 (0.8)	0 (0.0)	1 (1.0)
Anaemia	3 (0.5)	3 (1.8)	0 (0.0)	0 (0.0)	0 (0.0)
Ascites	3 (0.5)	0 (0.0)	0 (0.0)	0 (0.0)	2 (2.0)
Bowel obstruction	3 (0.5)	0 (0.0)	0 (0.0)	0 (0.0)	1 (1.0)
Haematemesis or haemoptysis	3 (0.5)	0 (0.0)	1 (0.8)	0 (0.0)	1 (1.0)
Cyanosis or hypoxia	2 (0.3)	1 (0.6)	0 (0.0)	0 (0.0)	0 (0.0)
Dysuria	2 (0.3)	1 (0.6)	0 (0.0)	0 (0.0)	0 (0.0)
Haematuria	2 (0.3)	0 (0.0)	1 (0.8)	0 (0.0)	0 (0.0)
Itch	2 (0.3)	2 (1.2)	0 (0.0)	0 (0.0)	0 (0.0)
Seizure	2 (0.3)	1 (0.6)	0 (0.0)	0 (0.0)	2 (2.0)
Urinary retention	2 (0.3)	1 (0.6)	0 (0.0)	0 (0.0)	0 (0.0)
Abdominal distention	1 (0.2)	1 (0.6)	0 (0.0)	0 (0.0)	0 (0.0)
Hypercalcaemia	1 (0.2)	1 (0.6)	0 (0.0)	0 (0.0)	0 (0.0)
Infection	1 (0.2)	1 (0.6)	0 (0.0)	0 (0.0)	0 (0.0)
Total	106 (18.4)	25 (15.3)	15 (11.5)	4 (10.0)	35 (34.7)

*PR = per rectum. PV = per vaginal.*

Additional palliative care symptoms were recorded in *n* = 106 (18.4%) of all analysed attendances (Table 2). Weight loss or cachexia, agitation or distress, and anaemia were the most recorded additional palliative care symptoms in consultations originally coded as other. Agitation or distress, acute neurological symptoms, dizziness, and coma or unconscious were the most recorded additional palliative care symptoms in consultations originally coded as missing. Additional palliative care symptoms most recorded in the free text for consultations originally coded as pain included dizziness, reflux or dysphagia, oedema, and weight loss or cachexia, and for consultations originally coded as palliative care included agitation or distress, oedema, ascites, dizziness, jaundice, seizure, and weight loss or cachexia.

### Evidence of anticipatory care planning

DNACPR forms were only discussed or addressed in a minority of GPOOH consultations; nothing in the coded or uncoded data established whether they were present or absent in 93.9% (*n* = 540) of attendances (Table 3). Only 12.6% of GPOOH attendances reflected the intention to use eKIS records; eKIS were recorded as being accessed in 55 (9.6%) attendances and recorded as absent in 3.0% of attendances. For the majority of GPOOH consultations, there was no record, either coded or free text, of whether eKIS had been considered by the attending clinician. JIC medication was recorded as having been prescribed in the free text for 65 (11.3%) attendances overall. For attendances originally coded as ‘palliative care’, 41 (40.6%) recorded that JIC medication was present before the consultation and 14 (13.9%) recorded that JIC medication was absent. JIC medication was more likely to be recorded absent in consultations originally coded as pain than in the baseline of all analysed attendances. In cases where JIC medication was recorded as being absent, they were prescribed by GPOOH during 20.5% (*n* = 9/44, all attendances) to 42.9% (*n* = 6/14, palliative care) of consultations. However, in 11.4% (*n* = 5/44, all attendances) to 14.3% (*n* = 2/14, palliative care) of consultations where JIC medication was absent, the attending clinician made a request for the patient’s own GP to prescribe JIC medication, rather than prescribing it during the consultation.

**Table 3.** Evidence of anticipatory care planning in free-text analysis: DNACPR forms, JIC medication, and Key Information Summaries

**Anticipatory care paperwork and medication**	**Incidence in all analysed free text (*n* = 575 attendances), *n* (%)**		**Incidence in attendances originally coded as ‘Other’ (*n*= 163), *n* (%)**		**Incidence in attendances originally coded as ‘Missing’ (*n* = 130), *n* (%)**		**Incidence in attendances originally coded as ‘Pain’ (*n* = 40), *n* (%)**		**Incidence in attendances originally coded as ‘Palliative care’ (*n*= 101), *n* (%)**	
**DNACPR forms and JIC medication**	**DNACPR form**	**JIC medication**	**DNACPR form**	**JIC medication**	**DNACPR form**	**JIC medication**	**DNACPR form**	**JIC medication**	**DNACPR form**	**JIC medication**
In place before attendance	16 (2.8)	65 (11.3)	3 (1.8)	12 (7.4)	0 (0.0)	2 (1.5)	0 (0.0)	2 (5.0)	8 (7.9)	41 (40.6)
Not in place before attendance	19 (3.3)	44 (7.7)	6 (3.7)	13 (8.0)	4 (3.1)	4 (3.1)	2 (5.0)	6 (15.0)	3 (3.0)	14 (13.9)
*No arrangements made for JIC*	—	30 (5.2)	—	10 (6.1)	—	0 (0.0)	—	0 (0.0)	—	6 (5.9)
*Prescribed during encounter*	—	9 (1.6)	—	1 (0.6)	—	0 (0.0)	—	0 (0.0)	—	6 (5.9)
*Regular GP asked to prescribe*	—	5 (0.9)	—	2 (1.2)	—	0 (0.0)	—	0 (0.0)	—	2 (2.0)
Unknown	540 (93.9)	466 (81.0)	154 (94.5)	138 (84.7)	126 (96.9)	124 (95.4)	38 (95.0)	32 (80.0)	90 (89.1)	46 (45.5)
**Electronic Key Information Summary (eKIS)**	**eKIS**		**eKIS**		**eKIS**		**eKIS**		**eKIS**	
Not mentioned	503 (87.5)		146 (89.6)		128 (98.5)		36 (90.0)		72 (71.3)	
Not present before attendance	17 (3.0)		5 (3.1)		1 (0.8)		2 (5.0)		4 (4.0)	
*No arrangements made*	14 (2.4)		4 (2.5)		1 (0.8)		1 (2.5)		4 (4.0)	
*GP asked to complete*	3 (0.5)		1 (0.6)		0 (0.0)		1 (2.5)		0 (0.0)	
Present and used in OOH consult	55 (9.6)		12 (7.4)		1 (0.8)		2 (5.0)		25 (24.8)	

*DNACPR = do not attempt cardiopulmonary resuscitation. JIC = just in case. OOH = out of hours.*

### Uncoded medication dispensed directly through GPOOH attendances

Medications dispensed through GPOOH were frequently recorded in the free text, rather than being recorded in the coded GPOOH prescribing section. In more than one-quarter (*n* = 157; 27.3%) of all consultations, analgesia was dispensed to the patient and recorded in free text without there being any coded prescribing record (Table 4). In more than half (*n* = 51; 50.5%) of consultations originally coded as palliative care, strong opioids and other drugs for symptom relief were dispensed to the patient and recorded in free text without there being any coded prescribing record. Uncoded prescriptions for medication for treatment for acute illnesses, including antibiotics, were recorded in the free text in one-sixth of all analysed consultations.

**Table 4.** Medication dispensed during consultation that was recorded in free text but not coded

**Drug name or drug category recorded in free text**	**Incidence in all analysed free text (*n* = 575 attendances), *n* (%)**	**Incidence in attendances coded as ‘Other’ (*n* = 163 attendances), *n* (%)**	**Incidence in attendances coded as ‘Missing’ (*n* = 130 attendances), *n* (%)**	**Incidence in attendances coded as ‘Pain’ (*n* = 40 attendances), *n* (%)**	**Incidence in attendances coded as ‘Palliative care’ (*n* = 101 attendances), *n* (%)**
Paracetamol	24 (4.2)	8 (4.9)	0 (0.0)	10 (25.0)	3 (3.0)
Non-steroidal anti-inflammatory drugs	6 (1.0)	4 (2.5)	0 (0.0)	1 (2.5)	0 (0.0)
Weak opioids	12 (2.1)	2 (1.2)	0 (0.0)	7 (17.5)	0 (0.0)
Strong opioids	106 (18.4)	25 (15.3)	2 (1.5)	13 (32.5)	51 (50.5)
Other analgesia	9 (1.6)	2 (1.2)	0 (0.0)	1 (2.5)	2 (2.0)
Other drug for symptom relief	139 (24.2)	37 (22.7)	1 (0.8)	8 (20.0)	51 (50.5)
Other drug for treatment for acute illnesses	96 (16.7)	27 (16.6)	3 (2.3)	7 (17.5)	7 (6.9)

## DISCUSSION

### Summary

For patients with advanced cancer, the prevalence of missing or uninterpretable (‘other’) clinical codes, used in routinely collected healthcare data, has resulted in previous studies, which have relied on coded GPOOH data, producing substantial underestimates of both the amount of pain and palliative care management delivered through the GPOOH service, and the level of multimorbidity and medical complexity addressed within these consultations.

Breathlessness, vomiting, cough, and nausea were the commonest key palliative care symptoms recorded in GPOOH consultation free text. Agitation or distress, oedema, and weight loss or cachexia were the commonest additional palliative care symptoms recorded in consultation free text. Anticipatory care planning, including DNACPR forms, eKIS, and JIC medication, was often absent; however, its presence or absence was poorly recorded in both coded and uncoded records. Analgesia for all consultations and other medications for symptom relief for attendances coded as palliative care were dispensed directly from GPOOH, without being recorded in the coded prescribing data, in more than one- quarter of analysed GPOOH consultations.

Meeting the complex needs of patients within the restrictions of an already overstretched unscheduled care service is a huge challenge, and one that has been exacerbated by the COVID-19 pandemic. The volume and complexity of palliative care delivered through GPOOH should be recognised as a priority for the GPOOH service and considered in future service design and delivery. Focus must be given to education, training, and workforce requirements.

### Strengths and limitations

To the authors’ knowledge, this is the first quantitative free-text analysis of uncoded GPOOH data in the UK. The complete availability of all coded and uncoded data for each GPOOH consultation was a significant strength, and vital to establishing this analysis as the most complete analysis of quantitative GPOOH data to date. The time intensity of reading free-text entries, and limited availability of suitable clinicians for reading, analysing, and assigning clinical codes to the GPOOH consultations, necessitated restricting the free-text analysis to only 10% of GPOOH consultations. Including a larger sample size would have increased the reliability of the free-text analysis. The age of the dataset was a limiting factor; however, no changes in how data are recorded in GPOOH have occurred since the data were gathered, and the underlying lessons are still relevant. Free-text analysis relies on individual interpretation of data, which can be subjective; however, this has been mitigated in this analysis by having reviewers undertake their assessments independently, and through kappa analysis to confirm strong agreement. This study was limited to palliative care presentations in a population of patients with cancer, and therefore did not include consultations for patients dying from non-cancer causes. Future studies should consider examining all palliative care delivered through GPOOH irrespective of underlying diagnosis.

### Comparison with existing literature

Pain or palliative care were the primary reasons for nearly half of all assessed GPOOH attendances. Previous studies on unscheduled care have also found that pain is the commonest presenting complaint at unscheduled care; however, the proportion of attendances found to be pain related varied widely with a range of 5%–83% of presentations.^14^^,^^29^^–^^40^ The present study’s finding that the proportion of attendances owing to pain was substantially higher on free-text analysis than on analysis of coded data alone suggests that variability in the completeness or accuracy of coded data may be responsible for this wide variation in reported levels of pain-related attendances. The high prevalence of key and additional palliative care symptoms in consultations coded as ‘pain’ suggests pain is frequently a signal symptom and that a holistic assessment is important to identify other underlying unmet palliative care needs. Patients without JIC medication in place were more likely to have pain- related attendances than those who had JIC medication prescribed, underscoring the importance of access to breakthrough analgesia in alleviating pain in patients with advanced cancer.

More than half of all GPOOH attendances recorded at least one key or additional palliative care symptoms. After pain and palliative care, respiratory tract infections, medication requests, vomiting, and breathlessness were the next commonest reasons for GPOOH attendance in this cohort. These findings are mirrored in previous research, where breathlessness^14^^,^^29^^–^^34^^,^^36^^,^^38^^,^^41^^–^^43^ and gastrointestinal symptoms^14^^,^^29^^–^^34^^,^^37^^,^^38^^,^^41^^,^^43^ are consistently among the commonest reasons for unscheduled care attendance by people with advanced cancer. Requiring prescribed medication has been reported to a lesser degree as being a reason for unscheduled care attendance in people with advanced cancer.^29^^,^^44^^,^^45^ This analysis found that the proportion of consultations for breathlessness or lower respiratory tract infections, and medication requests was higher on free-text analysis than on coded data, and higher still when considering additional palliative care symptoms recorded in free text but which were not the patient’s main reason for presentation. Breathlessness, for example, was the original presenting complaint in 2.3% (*n* = 13) of attendances, the assigned presenting complaint in 5.2% (*n* = 30) of attendances, and a key palliative care symptom in 20.2% (*n* = 116) of attendances. The prevalence of these symptoms is likely to be greater than is reported in existing literature.

This analysis found that, for patients with advanced cancer, uncoded medication for analgesia was dispensed or administered in more than one-quarter of all GPOOH consultations. In addition, in half of palliative care consultations, uncoded medication for strong opioids and other drugs for symptom relief were dispensed or administered. While some of these medications were administered from the patient’s own stock of regular or JIC medication, many were distributed directly from GPOOH, and uncoded. This suggests that the need for medication is a previously under-reported and under-acknowledged factor driving GPOOH consultations for patients with advanced cancer. This emphasises the importance of access to a wide range of stock medication for GPOOH services, and the access to pharmacy provision out of hours to support patients with advanced cancer.

### Implications for research and practice

This research identifies the substantial amount of information missed by large data analysis using coded healthcare data in GPOOH and suggests that current literature considerably underestimates the breadth and depth of palliative care delivered through GPOOH. This analysis demonstrates that the majority of GPOOH attendances involve holistically managing multiple pain and palliative care symptoms. It emphasises the importance of GPOOH in providing effective community palliative care and highlights the importance of GPOOH practitioners being trained, experienced, skilled, and motivated in providing high-quality palliative care. The frequency with which medication, particularly analgesia, was dispensed through GPOOH suggests that improving in-hours provision of anticipatory and JIC medication may play a role in improving patients’ symptom burden and minimising potentially avoidable GPOOH attendances. Improved handovers from in-hours GPs to GPOOH, and increased integration with in-hours and out-of-hours specialist palliative care services, may help to address some of the anticipatory planning and medication provision gaps in care identified in this analysis.

Improving data-capturing processes during healthcare consultations is essential to improve the accuracy and interpretability of routine healthcare datasets. Such adaptations could be achieved through software changes, including disabling the ability to input ‘other’ or ‘missing’ clinical codes, and improving automatic capture of information such as eKIS and DNACPR use. Additional training for clinicians in the importance and uses of coded data may also improve the accuracy and completeness with which consultation data are recorded. Future research to characterise delivery of care and patient experience of GPOOH should include qualitative and mixed- methods methodologies to avoid solely relying on large dataset analyses. Such research is vital to achieving a more complete understanding of patients’ experiences of GPOOH, and to inform the future design of this service.

In conclusion, a substantial amount of the information contained within GPOOH consultations is not coded and is therefore uninterpretable in quantitative analysis of large healthcare datasets. Pain and palliative care symptoms are common reasons for attendance at GPOOH by people with advanced cancer; the care they receive through GPOOH is multidimensional and complex. Analyses relying solely on coded data risk substantially under-reporting the volume and complexity of pain management and palliative care delivered in GPOOH.

## Data

The general practice out-of-hours dataset that was built and analysed during this research is not publicly available owing to its use of de-identified but individual-level healthcare data. Access is available through the Health Informatics Centre, Dundee, via the corresponding author on reasonable request and subject to Caldicott Guardian approval.
